# Painful ophthalmoplegia with normal cranial imaging

**DOI:** 10.1186/1471-2377-14-7

**Published:** 2014-01-09

**Authors:** Chih-Hsien Hung, Kuo-Hsuan Chang, Chun-Che Chu, Ming-Feng Liao, Hong-Shiu Chang, Rong-Kuo Lyu, Yi-Ming Wu, Yao-Liang Chen, Chiou-Lian Lai, Hsiao-Jung Tseng, Long-Sun Ro

**Affiliations:** 1 Department of Neurology, Kaohsiung Municipal Hsiaokang Hospital, Kaohsiung, Taiwan; 2 Department of Neurology, Kaohsiung Medical University Chung-Ho Memorial Hospital; Kaohsiung Medical University, Kaohsiung, Taiwan; 3 Department of Neurology, Linkou Campus, Chang Gung Memorial Hospital and University College of Medicine, Gueishan Township, Taiwan; 4 Department of Radiology, Linkou Campus, Chang Gung Memorial Hospital and University College of Medicine, Gueishan Township, Taiwan; 5 Department of Radiology, Keelung campus, Chang Gung Memorial Hospital and University College of Medicine, Keelung City, Taiwan; 6 Biostatistical Center for Clinical Research, Chang Gung Memorial Hospital, Gueishan Township, Taiwan

**Keywords:** Headache, Ocular diabetic neuropathy, Ophthalmoplegic migraine, Painful ophthalmoplegia, Tolosa-Hunt syndrome

## Abstract

**Background:**

Painful ophthalmoplegia with normal cranial imaging is rare and confined to limited etiologies. In this study, we aimed to elucidate these causes by evaluating clinical presentations and treatment responses.

**Methods:**

Cases of painful ophthalmoplegia with normal cranial MRI at a single center between January 2001 and June 2011 were retrospectively reviewed. Diagnoses of painful ophthalmoplegia were made according to the recommendations of the International Headache Society.

**Results:**

Of the 58 painful ophthalmoplegia cases (53 patients), 26 (44.8%) were diagnosed as ocular diabetic neuropathy, 27 (46.6%) as benign Tolosa-Hunt syndrome (THS), and 5 (8.6%) as ophthalmoplegic migraine (OM). Patients with ocular diabetic neuropathy were significantly older (62.8 ± 7.8 years) than those with benign THS (56.3 ±12.0 years) or OM (45.8 ± 23.0 years) (*p* < 0.05). Cranial nerve involvement was similar among groups. Pupil sparing was dominant in each group. Patients with benign THS and OM responded exquisitely to glucocorticoid treatment with resolved diplopia, whereas patients with ocular diabetic neuropathy didn’t (*p* < 0.05). Patients with OM recovered more rapidly than the other groups did (*p* < 0.05). Overall, most patients (94.8%) recovered completely during the follow-up period.

**Conclusions:**

Ocular diabetic neuropathy and benign THS accounted for most of the painful ophthalmoplegias in patients with normal cranial imaging. Patient outcomes were generally good.

## Background

Painful ophthalmoplegia consists of periorbital or hemicranial pain with ipsilateral ocular motor nerve palsies [1]. The syndrome involves diverse causes; therefore, a comprehensive evaluation is essential. Contrast-enhanced magnetic resonance imaging (MRI) provides sensitive detection of structural abnormalities, such as trauma, infection, malignancy, or vascular anomaly [2,3].

However, neuroimaging studies frequently yield negative findings in patients with painful ophthalmoplegia [4]. Cranial neuropathies in diabetic patients due to microvasculopathy occasionally demonstrate painful ophthalmoplegia with normal neuroimaging studies [5,6]. Maintaining optimal glycemic control may aid recovery [5]. Ophthalmoplegic migraine (OM), which frequently occurs in childhood [7], may also present as painful ophthalmoplegia in adulthood upon normal imaging studies [8]. The use of glucocorticoids along with migraine prophylaxes, such as β-blockers or calcium channel blockers, may hasten recovery [8]. Occasionally, idiopathic inflammation of the cavernous sinus (CS) can cause painful ophthalmoplegia. Tolosa-Hunt syndrome (THS) is caused by an inflammatory process of unknown etiology. Although granulomatous tissues in the CS can often be identified in these patients (referred to as inflammatory THS), half of the patients with clinical presentation and diagnosis of THS have no radiographic evidence of inflammation [9,10]. The eponym “benign THS” has been introduced for patients with THS and normal neuroimaging findings [9]. Glucocorticoids are considered as standard treatment for patients with THS [1,10].

Given that the choice of treatment depends on an accurate diagnosis, it is essential to understand the clinical presentations in patients with MRI-negative painful ophthalmoplegia. In this study, we aimed to elucidate the causes in this group of patients by evaluating their clinical presentations and responses to treatment. These results will be important for guiding appropriate treatment decisions in patients with painful ophthalmoplegia.

## Methods

Consecutive adult patients referred with painful ophthalmoplegia to Chang Gung Memorial Hospital were collected from January 2001 to June 2011. Their in-hospital medical chart records were retrospectively reviewed with approval of the Institutional Review Board of the Chang Gung Memorial Hospital (license ID: 100-0136B). Patients who developed periorbital or hemicranial pain combined with ipsilateral oculomotor, trochlear, and/or abducent palsies were recruited in this study. All patients had appropriate laboratory workup, including complete hematologic and routine tests, electrolytes, renal and liver function test, anti-nuclear antibody, thyroid function, C-reactive protein, elevated sedimentation rate (ESR), fasting glucose, and glycohemoglobin (HbA1c). All recruited patients also underwent cranial MRI and cerebral vascular investigations (MR angiography or digital subtraction angiography). None of the patients had concurrent infection, malignancy, Sjögren’s syndrome, systemic lupus erythematosus, rheumatoid arthritis, vasculitis, cranial herpes zoster, Horton arteritis, or immunocompromised status.

The diagnosis of painful ophthalmoplegia was made according to the recommendations of the International Headache Society (IHS), 2^nd^ edition (ICDH-2) [4]. Patients with diabetes (fasting plasma glucose ≥ 126 mg/dL or HbA1c ≥ 6.5%) developing painful ophthalmoplegia without other attribution after workup were designated as ocular diabetic neuropathy [4,11]. Patients with idiopathic unilateral painful ophthalmoplegia fulfilling the IHS criteria for THS and with normal cranial imaging were designated as benign THS [9]. Since THS symptoms may resolve spontaneously when left untreated [1,10,12], patients with idiopathic painful ophthalmoplegia fulfilled THS criteria (A, B and C) and self-remission within 3 months were also considered as those with THS. These patients with THS may have delayed diplopia improvement after 6–8 weeks of glucocorticoid treatment [13]; therefore, patients fulfilling the THS criteria with pain resolving within 72 hours after treatment, but without immediate improvement in their diplopia, were also allocated to the THS group. Patients with migrainous headache accompanied or followed by ophthalmoplegia within 4 days in accordance with the IHS criteria for OM was allocated to the OM group [4]. Patients with a single attack fulfilling the OM criteria were also considered as having OM [8,14]. All enrolled patients underwent a follow-up period of at least 6 months to ensure no other etiology was identified.

All enrolled patients underwent cranial MRI with a standard head coil on a 1.5- to 3-T MR scanner. The predefined MR scan parameters used in this study were as follows: TR = 450–550 ms, TE = 9.9–12 ms, field of view (FOV) = 200 × 200 mm, and matrix = 512 × 512 in transverse and coronal T1-weighted spin-echo sequences before and after intravenous administration of gadolinium enhancement (0.2 ml/kg); TR = 3114–4000 ms, TE = 81–90 ms, FOV = 200 × 200 mm, matrix = 512 × 512 in transverse T2 fast spin-echo sequence; and TR = 6000–9000, TE = 105–142 ms, FOV = 200 × 200 mm, and matrix = 512 × 512 for transverse fluid-attenuated inversion recovery sequence (FLAIR). Sections were either 3 or 5 mm in thickness. Cerebral vascular investigations, including MR angiography or digital subtraction angiography, were performed in all recruited patients to exclude vascular anomalies.

Cranial scans of painful ophthalmoplegias were individually and retrospectively reviewed, by 2 experienced neuroradiologists (Wu, YM and Chen, YL) with a focus on the parasellar area and orbits. Both reviewers were aware of the symptom of painful ophthalmoplegia but were blind to the final diagnosis. Only cases with normal neuroimaging findings, consented by both reviewers, were further analyzed.

Glucocorticoid dosages and response to treatment were registered. A positive response to glucocorticoids was defined as relief of symptoms or improved neurologic signs within 3 days of glucocorticoid treatment initiation [4,15,16].

Statistical analyses were performed using SPSS statistical software (version 17.0, Chicago, IL, USA). Descriptive statistics were expressed mean ± standard deviation (SD) for continuous data, whereas the number with its percentage for categorical data. Continuous variables were compared using Kruskal Wallis tests followed by post-hoc Dunn tests if statistically significant. Categorical variables were compared using chi-square test. Disease course was estimated according to the Kaplan-Meier method. The endpoint of the primary study was the time until complete resolution of symptoms and signs. All p values were two-tailed. In all tests, p < 0.05 was considered statistically significant.

## Results

**Figure 1:**
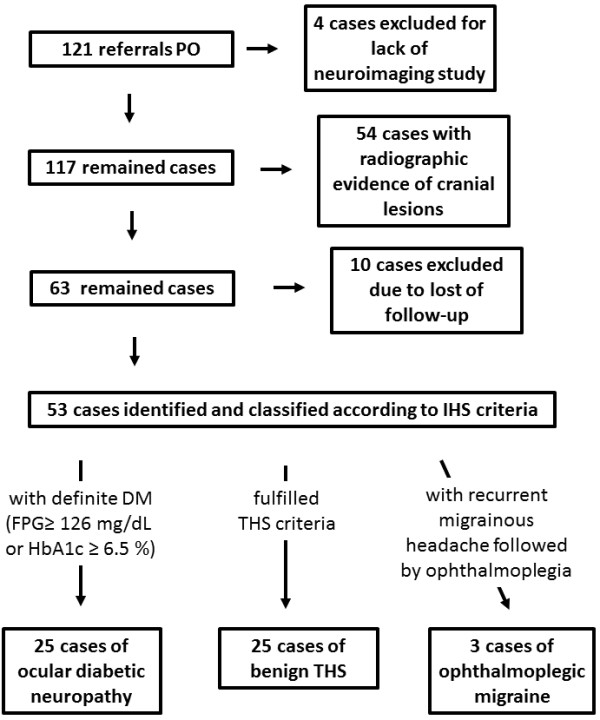
**Flow chart illustrating the diagnostic process of painful ophthalmoplegias with normal cranial imaging and the diagnostic outcome after investigation.** (Abbreviations: PO = painful ophthalmoplegia; DM = diabetes mellitus; FPG = fasting plasma glucose; HbA1C = glycohemoglobin; THS = Tolosa-Hunt syndrome).

There were 121 referrals with symptomatic diagnoses of unilateral painful ophthalmoplegia (Figure 1). Four patients were excluded because of incomplete neuroimaging and/or cerebral vascular investigations. Of the remaining 117 patients, 54 patients with radiographic evidence of cranial lesions were excluded because of the following conditions: carotid-cavernous fistula (10 patients), P-com aneurysm (2 patients), primary head and neck neoplasms (6 patients), primary intracranial neoplasm (3 patients), cavernous sinus metastases (2 patients), sinonasal infection (5 patients), orbital infection (4 patients), trauma (1 patient), and idiopathic inflammatory Tolosa-Hunt syndrome (21 patients). Ten patients with initial normal neuroimaging were also excluded because of the lack of follow-up. Finally, 53 qualified patients with normal neuroimaging data were recruited for this study. Twenty-five patients (47.2%) were diagnosed as having ocular diabetic neuropathy, and 25 (47.2%) and 3 (5.7%) patients were diagnosed with benign THS and OM, respectively (Table 1). Recurrent episodes were reported in 1 patient (4.0%) with ocular diabetic neuropathy, 1 patient (33%) with OM, and 2 patients (8.0%) with benign THS. A total of 58 episodes were recruited for further analysis.

**Table 1:** Demographic data and clinical manifestations of painful ophthalmoplegia with normal neuroimaging

	**Ocular diabetic neuropathy**	**Benign Tolosa-Hunt syndrome**	**Ophthalmoplegic migraine**	**Total**	***p *** **value**
**N (%)**	25 (47.2)	25 (47.2)	3 (5.7 )	53	
**N of episodes**	26	27	5	58	
**M/F**	12/13	10/15	3/0	25/28	
**Age (year)**	62.8 ± 7.8	56.3 ± 12.0	45.8 ±23.0	58.3 ± 12.4	0.008*
**Involvement of cranial nerves, N (%)**					
CN3	19 (73.1)	20 (74.1)	4 (80.0)	43 (74.1)	0.949
CN4	9 (34.6)	15 (55.6)	2 (40.0)	23 (39.7)	0.301
CN6	13 (50.0)	16 (59.3)	2 (40.0)	31 (53.4)	0.652
Ptosis	16 (61.5)	11 (40.7)	1 (20.0)	28 (48.3)	0.132
Pupillary dysfunction	7 (26.9)	6 (22.2)	0 (0.0)	13 (22.4)	0.417
Multiple CNs involvement	10 (38.5)	18 (66.7)	3 (60.0)	31 (53.4)	0.115
**Laboratory data**					
ESR (mm/hr)	29.4.2 ± 28.0	14.2 ± 21.7	9.6 ± 8.4		0.070
HbA1c (%)	8.3 ± 1.8	5.9 ± 2.5	6.2 ± 0.6		0.003*
**Recurrence, N of patients (%)**	1 (4.0)	2 (8.0)	1 (33.3)		0.190
**Duration of course (month)**	2.0 ± 1.0	1.6 ± 0.8	0.9 ± 0.4	1.7 ± 0.9	0.044*
**Duration of follow-up (year)**	3.4 ± 3.1	4.9 ± 3.6	3.6 ± 0.3	4.0 ± 3.3	
**Total recovery, N (%)**	24 (92.3)	26 (96.3)	5 (100.0)	55 (94.8)	0.695

Symbols and abbreviations: *N* number; *CN* cranial nerve; *ESR* elevated sedimentation rate; *HbA1c* glycohemoglobin. Footnotes: * = statistically significant value.

Demographic data and clinical presentations between the groups are summarized in Table 1. Patients with ocular diabetic neuropathy were significantly older than patients with benign THS (post-hoc tests; *p* = 0.045) and patients with OM (*p* = 0.004) (Table 1). All patients with diabetic ophthalmoplegia had type II diabetes mellitus (DM), and the known duration of diabetes was 9.9 ± 9.4 years. Five patients (20.0%) were newly diagnosed with DM. No patient with final diagnosis of THS or OM had diabetes or impaired fasting glucose. All patients with OM had adult-onset migrainous headache.

Cranial nerve involvement was not significantly different among groups (Table 1). The most frequently involved cranial nerve was the oculomotor nerve in all groups (74.1%), followed by the trochlear nerve (53.4%) and the abducens nerve (39.7%). Pupillary function was not altered in most patients; pupil sparing was common in patients with ocular diabetic neuropathy as well as in other groups. Compared to patients with diabetic ophthalmoplegia, those with benign THS and OM were prone to multiple ocular motor neuropathies, even though there was no significant difference among groups.

In the laboratory examinations, the ESR was not significantly different within groups. Anti-nuclear antibody (ANA) and rheumatoid factor (RF) were also non-specific. Compared to benign THS (5.9 ± 2.5%) and OM (6.2 ± 0.6%), ocular diabetic neuropathy was associated with higher levels of glycohemoglobin (8.4 ± 1.8%, *p* = 0.003).

All enrolled patients underwent a follow-up period of at least 6 months to ensure no other etiology was identified. The mean duration from initial symptoms to the last follow-up was 3.4, 4.9, and 3.6 years in patients with ocular diabetic neuropathy, benign THS, and OM, respectively. No patients had newly diagnosed autoimmune disease, intracranial malignancy, vascular malformation, sarcoidosis, central nervous system (CNS) infection, or HIV infection during the follow-up period.

**Figure 2:**
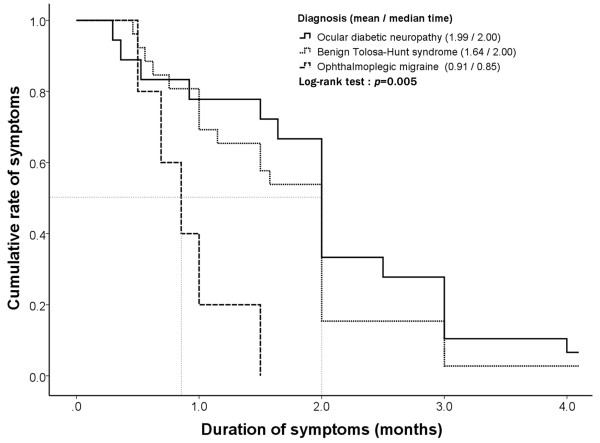
**The clinical courses of ocular diabetic neuropathy, benign Tolosa-Hunt syndrome (THS), and ophthalmoplegic migraine (OM) are illustrated.** The primary study endpoint was the time to complete resolution of painful ophthalmoplegia. The duration of clinical course differed among these groups (Log-rank test, p = 0.005). OM (mean = 0.91 months) showed a significantly more rapid recovery than benign THS (mean = 1.64 months) and ocular diabetic neuropathy (mean = 1.99 months). Overall, most of the patients recovered completely (94.8%).

The duration from onset to complete recovery in ocular diabetic neuropathy was 2.0 ± 1.0 months, which was similar to that in benign THS (1.6 ± 0.8 months). Recovery was significantly more rapid (0.9 ± 0.4 months) in OM than either in benign THS or in ocular diabetic neuropathy (log-rank test, *p* = 0.005, Figure 2). In most cases (n = 55, 94.8%), patients reported complete recovery within 3 months, except for 1 case of benign THS (3.7%) and 2 cases of ocular diabetic neuropathy (8.3%) with minor sequelae of diplopia.

Glucocorticoids were prescribed in 24 episodes (88.9%) of benign THS and 4 of OM (80.0%). Therapeutic trial of glucocorticoids was used in 13 episodes (50.0%) of ocular diabetic neuropathy because of initially inconclusive diagnoses. Four patients with benign THS and 2 patients with ocular diabetic neuropathy improved before glucocorticoid use and, thus, were excluded from these analyses. The mean dose of prednisolone was 1.3 ± 3.7 mg/kg/day.

Immediate pain relief within 72 hours of treatment was observed in nearly all patients. Regarding resolved diplopia, patients with benign THS and OM responded well to glucocorticoids (95.0% and 100.0%, respectively), but only partial patients with ocular diabetic neuropathy (63.6%) did so (p = 0.040, Table 2).

**Table 2:** Response rate of pain and diplopia to glucocorticoids in cases of painful ophthalmoplegia with normal neuroimaging

**Response to glucocorticoids**	**Ocular diabetic neuropathy**	**Benign Tolosa-Hunt syndrome**	**Ophthalmoplegic migraine**	**Total**	***p *** **value**
**Pain, N (%)**	10 (90.9)	20 (100.0)	4 (100.0)	34 (97.1)	0.325
**Diplopia, N (%)**	7 (63.6)	19 (95.0)	4 (100.0)	30 (85.7)	0.040

A positive response to glucocorticoids was defined as relief of symptoms or improvement of neurologic signs within 3 days of glucocorticoid treatment. MRI = magnetic resonance imaging; N = number.

## Discussion

The current study has several findings. Benign THS and ocular diabetic neuropathy accounted for the majority of cases of painful ophthalmoplegia with normal cranial imaging (91.4%). Clinical presentations were similar between the groups, except for the age of onset, response to glucocorticoids, and duration of disease course. Nearly all patients with benign THS and OM had an immediate improvement in ocular motor function after initiation of glucocorticoid treatment, whereas only partial patients with diabetic ophthalmoplegia did so. Patients with OM recovered more rapidly than the other groups did. Overall, most patients recovered completely during the follow-up period.

Patients with ocular diabetic neuropathy were significantly older than patients with benign THS and OM. This result is in accordance with previous reports; the mean reported age of diabetic ophthalmoplegias ranges from 50.2 to 68 years [6,17-19], which is more than that of benign THS (48.6 years) and adult OM (36.4 years) [8,9]. Diabetic neuropathy is predominantly a disease of older adults [20], whereas OM predominantly affects children and younger adults [7,8], and THS may present at any age [1,9].

Glucocorticoid administration is of therapeutic and partial diagnostic utility. The response of diplopia resolving was of higher diagnostic value than that of pain relieving. Orbital pain was dramatically relieved after glucocorticoid treatment in almost all patients. Regarding diplopia resolving, only THS and OM respond exquisitely to glucocorticoid therapy [1,7] The immediate improvement of ocular motor nerve dysfunction might reflect the inflammatory pathogenesis of these diseases [7,12,21,22]. In contrast, ischemic pathogenesis associated with ocular diabetic neuropathy [5,23,24] decreases the response to glucocorticoids, even though glucocorticoids can partially relieve pain caused by ischemic injury.

Patients with OM recovered faster than patients with THS and ocular diabetic neuropathy, consistent with previously reported results [7,10,18,19,25,26]. The average recovery time of OM is approximately 3 weeks, whereas that of THS is approximately 2 months and ocular diabetic neuropathy is 3–4 months [7,9,10,18,19]. Although the disease course varied among groups, the outcomes were generally good. In our experience, most patients recovered completely within 3 months. Physicians should be more vigilant if a patient fails to improve within 3 months, and further aggressive investigation may be indicated.

Pupil sparing was not only common in patients with ocular diabetic neuropathy, but was also common in patients with THS and OM. It is widely accepted that pupillary dysfunction is suggestive of aneurysmal or neoplastic compressive lesions [27,28], and it is not common in diabetic ophthalmoplegia [25,26,29]. Only 14–18% of patients with diabetic ophthalmoplegia develop pupillary dysfunction [29]. In addition, the pupil is occasionally affected in THS [4]. Although a majority of child patients with OM have pupillary involvement [7], adult patients with OMs tend to spare pupillary response [8]. Pupil sparing can be used as a potential marker for differentiating these ophthalmoplegias from those caused by structural compressive lesions.

Occasional reports have documented an elevated ESR in the acute stage of THS [1]. However, there is no convincing evidence for a correlation between connective tissue disease and THS. In our study, the ESR results were grossly normal in patients with benign THS. Therefore, ESR is of no diagnostic value in differentiating THS from other causes.

In the past, the absence of radiographic evidence of inflammation in patients with THS was confusing to clinicians, and a diagnosis of “idiopathic painful ophthalmoplegia” was applied to these patients [30-32]. The eponym of “benign THS” was first introduced by La Mantia et al. in 2006 to describe these patients [9]. According to the review by La Mantia et al., half of the THS patients manifest as benign condition. Given the nosography of benign THS has been proposed for less than a decade, it is probable that such cases will continue to be diagnosed. The exact proportion of benign and inflammatory cases remains to be determined. A strong similarity between benign and inflammatory THS was recently identified, and inflammation may contribute to the pathogenesis of both conditions [10]. An official definition and elaboration of benign THS is essential for clinical practice.

Painful ophthalmoplegia usually mandates an extensive systemic workup for an underlying neoplastic, inflammatory, infective or autoimmune disease. Because of the limitations of a retrospective study, an exhaustive checkup for screening was not possible. However, all recruited patients had ancillary laboratory workup, cranial MR imaging, and cerebral vascular investigation. In addition, none of the patients had newly diagnosed malignancies, autoimmune disease, or CNS infection during the follow-up period. Furthermore, the results were generally favorable during the follow-up period and all patients completely recovered or experienced minor sequelae. Therefore, after the follow-up, we could conclude that other causes of painful ophthalmoplegia were less likely and further confirmed the suitability of our diagnoses.

In this study, the classification was based on the ICHD-2 criteria and clinical specificity of each disease. Patients with definite diabetes (fasting plasma glucose ≥ 126 mg/dL or HbA1c ≥ 6.5%) were designated as ocular diabetic neuropathy [11]. In fact, it is difficult to differentiate ocular diabetic neuropathy from benign THS comorbid with diabetes or impaired glucose tolerance at this present technological stage. The limitation of the diagnostic criteria might lead to selection bias in the classification.

Diabetic neuropathy may manifest before the diagnosis of DM [5]. It is possible that patients manifesting ophthalmoplegia with undiagnosed DM could be mistaken as those with THS. However, diabetic cranial neuropathies mainly occur in older individuals with a long duration of diabetes (mean = 8.5 ~ 16 years) [6,17,19]; an occult diagnosis of diabetes is very less likely at this stage. In addition, none of the THS or OM patients in this study had a diagnosis of diabetes mellitus (fasting plasma glucose ≥ 126 mg/dL or HbA1c ≥ 6.5%) or impaired fasting glucose (fasting plasma glucose ≥ 100 mg/dL) [11]. Due to the limitation of a retrospective study, the data of impaired glucose tolerance (IGT), tested by oral glucose tolerance test, were incomplete in THS patients. Further prospective studies are warranted to clarify the relationship of benign THS and glucose intolerance.

Our study also had other limitations. First, the sample size of each group, especially the OM group, might be too small for adequate comparisons. Second, the conclusion that steroid is of partial diagnostic utility might result from the selection bias caused by the diagnostic criteria. Third, the retrospective analysis limited our ability to collect detailed information, forcing a reliance on documented findings of examinations. Fourth, histological examinations for pathological diagnoses lacked. Nevertheless, this study provides useful information about the clinical features of painful ophthalmoplegia and may warrant large-scale prospective studies to assess the optimal treatment approach.

## Conclusions

In summary, ocular diabetic neuropathy and benign THS account for most of the painful ophthalmoplegias in patients with normal cranial imaging. Glucocorticoid administration is of therapeutic and partial diagnostic utility because THS and OM respond exquisitely to the treatment. Patients with OM recover more rapidly than the other groups do. Patient outcomes are generally good.

## Consent

The Institutional Review Boards of the Chang Gung Memorial Hospital waived the need for individual informed consent because all data were anonymized and de-identified prior to analysis.

## Competing interests

The authors declare that there is no conflict of interests.

## Authors’ contributions

CH: acquisition, analysis and interpretation of data, drafting the manuscript. KC: analysis and interpretation of data. CC, ML, HC, RL and CL: substantial contributions to conception and design. YW and YC: reviewing and evaluating cranial images. HT: interpretation of data. LR: revising manuscript critically for important intellectual content. All authors read and approved the final manuscript.

## Pre-publication history

The pre-publication history for this paper can be accessed here:

http://www.biomedcentral.com/1471-2377/14/7/prepub
